# Pre-residency neurosurgical fellowship programs impact on a successful re-application: a departmental experience

**DOI:** 10.1007/s10143-025-03444-x

**Published:** 2025-03-12

**Authors:** Mareshah N. Sowah, Benjamin R. Klein, Victor M. Lu, Ricardo J. Komotar, Allan D. Levi

**Affiliations:** 1 University of South Carolina School of Medicine Greenville, Greenville, SC USA; 2 University of Toledo College of Medicine and Life Sciences, Toledo, Ohio USA; 3 Department of Neurological Surgery, University of Miami Miller School of Medicine, Lois Pope Life Center, 1095 NW 14th Terrace (D4-6), Miami, FL 33136 USA

**Keywords:** Neurosurgery, Pre-residency, Fellowship, International medical graduate, Match

## Abstract

Matching into neurosurgery residency within the United States is one of the most competitive endeavors for medical students. Pursuing a neurosurgery pre-residency fellowship program is becoming a popular option among domestic applicants, as well as international medical graduates (IMGs), who are unsuccessful in their neurosurgery match or wish to create a more competitive application prior to applying. The aim of this study was to review the University of Miami’s pre-residency fellowship program experience to date. Records were retrospectively reviewed for all pre-residency fellows that rotated within the Department of Neurosurgery at the University of Miami between 2000 (inception) to 2024 with match success rate as the primary outcome of interest. A total of 23 pre-residency fellows who trained within the University of Miami’s Department of Neurosurgery since the inception of the program were identified during the study period of 2000 to 2024. There were 15 (65%) IMGs and 8 (35%) United States medical graduates based on previous medical education. All of the fellows successfully completed their pre-residency training, and 12 (53%) successfully matched into neurosurgery. Another significant trend noted was that IMGs had more research and post-graduate neurosurgical experiences compared to US medical graduates at the time their fellowship began. Pre-residency fellowship programs are a feasible and tangible alternative route for neurosurgery match applicants who wish to augment their application. The outcomes of our pre-residency fellowship are promising, particularly for IMGs, however more prospective data and experiences across multiple departments are required to truly understand parameters of success for pre-residency fellowship programs in neurosurgery.

## Clinical trial number

Not applicable.

## Introduction

Residency training is the next step for medical students as they graduate from their respective programs and enter the field as practicing physicians. To begin residency training, medical students first have to “match” into a field of preference based on what interests them in medical school. Neurosurgery is a historically competitive field for a medical student to pursue. The 2024 “Advanced Data Tables” provided by the NRMP describe 116 neurosurgical residency programs, with 241 PGY-1 positions available at the beginning of the cycle [1]. There were 423 applicants to the specialty in 2024, with 299 MD seniors comprising the pool [1]. 204 of the 299 MD seniors (68%) matched into a neurosurgical residency program, and MD seniors composed 84.6% of the total 241 PGY-1 positions offered in the field [1]. In the past 4 years, 7 total additional PGY-1 positions have been added, and the trend of total MD seniors matching yearly into neurosurgery has been stable since 2020, with approximately 200 applicants per year [1].

As such, the majority of MD seniors applying into neurosurgery match into the field, but those that do not must either choose to pursue a different field of medicine or reapply the following cycle. International medical graduates (IMGs) have an even more challenging road, with historically low match rates into a US neurosurgical residency [2]. IMGs are disadvantaged when applying to neurosurgery residency. Some of the ways this is reflected is increased difficulty for IMGs to secure an audition elective/rotation, proper letters of recommendation especially from same specialty mentors, as well as cultural differences that may be present [3]. For example, the culture shift that IMGs may experience can be slight if an individual is acclimated to US customs and habits, but could potentially be more difficult for those who are unfamiliar with US customs or may not be fluent or conversational in English. In 2013, Yekula et al. analyzed IMG neurosurgery residency applicants and their outcomes and found that the acceptance rate was only 3.5% [2]. This number increased over a 7-year period with the 2020 acceptance rate for IMGs being 7.5%, implicating a 6.8% increase in likelihood of an IMG being accepted into neurosurgery residency per year.

For applicants who choose to reapply to neurosurgery, there are avenues to increase competitiveness such as neurosurgery pre-residency fellowships, ACGME general surgery internship years, and pursuing a neurosurgical research year. A pre-residency fellowship program in neurosurgery provides US medical graduates and IMGs with clinical and research experience necessary to be a successful neurosurgical resident. The goal of such programs is to gain hands-on neurosurgical clinical experience by being directly involved in the treatment and care of neurosurgical patients. Specifically, pre-residency fellows manage floor and ICU patients, see consults, scrub-in to neurosurgical cases, take call, and participate in research. Currently, pre-residency neurosurgical fellowships are offered at Boston Medical Center, Lahey Hospital and Medical Center, Icahn School of Medicine at Mount Sinai, University of New Mexico Health Sciences Center, Mayo Clinic College of Medicine and Science - Rochester The University of Minnesota, The University of Iowa, and The University of Miami Miller School of Medicine. To date, no such manuscript that explores factors and outcomes of fellows from these different programs exists. In this study, we analyze the University of Miami’s pre-residency neurosurgery fellowship program and the ultimate match outcomes. This information is pertinent to providing insight into the success of pre-residency neurosurgical fellowship and the ultimate goal of matching into a neurosurgery residency program.

## Materials & methods

### Cohort

Administrative records were retrospectively reviewed for all pre-residency fellows that rotated within the Department of Neurosurgery at the University of Miami during the study period of 2000 to 2024. Pre-residency fellows were defined as medical school graduates undergoing neurosurgical clinical experiences equivalent to the level of a PGY-1 to PGY-7. Pre- and post-fellowship data were evaluated to determine success and outcomes following enrollment in the pre-residency fellowship. An IMG was defined as any resident who had completed their primary medical degree at a medical institution outside of the United States.

### Outcomes of interest

The primary outcome of interest for this study was the ability to successfully match into neurosurgical residency following completion of the pre-residency fellowship at the University of Miami. Secondary outcomes of interest included geographical distribution of matched applicants, pre-fellowship metrics (clinical exposure and research experience), and country of origin.

## Results

### Full cohort characteristics

**Fig. 1:**
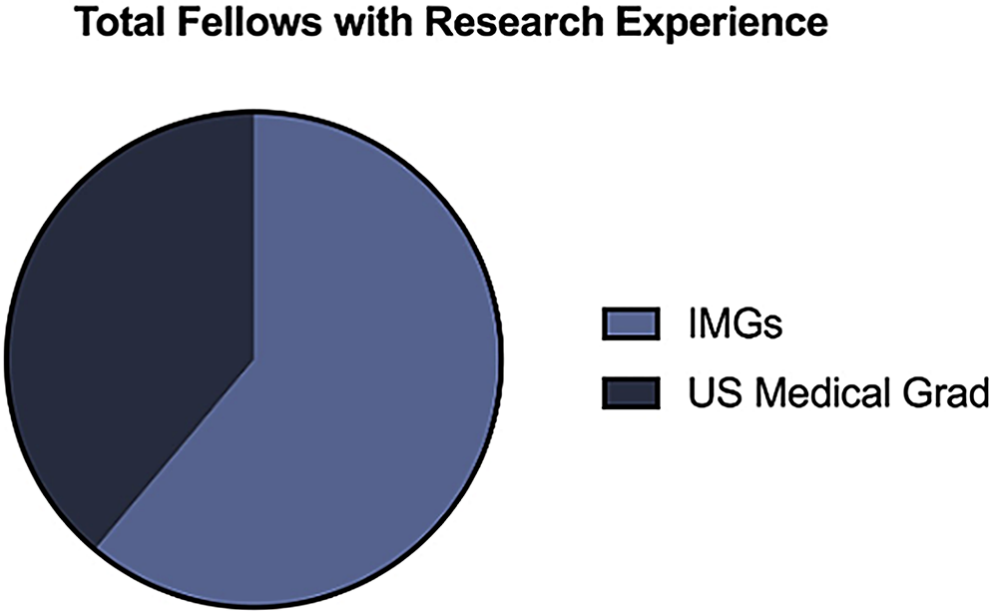
Graph depicting prior research exposure prior to acceptance into the pre-residency fellowship program, differentiated between international medical graduates (IMGs) and U.S. medical graduates (*n* = 18)

**Fig. 2:**
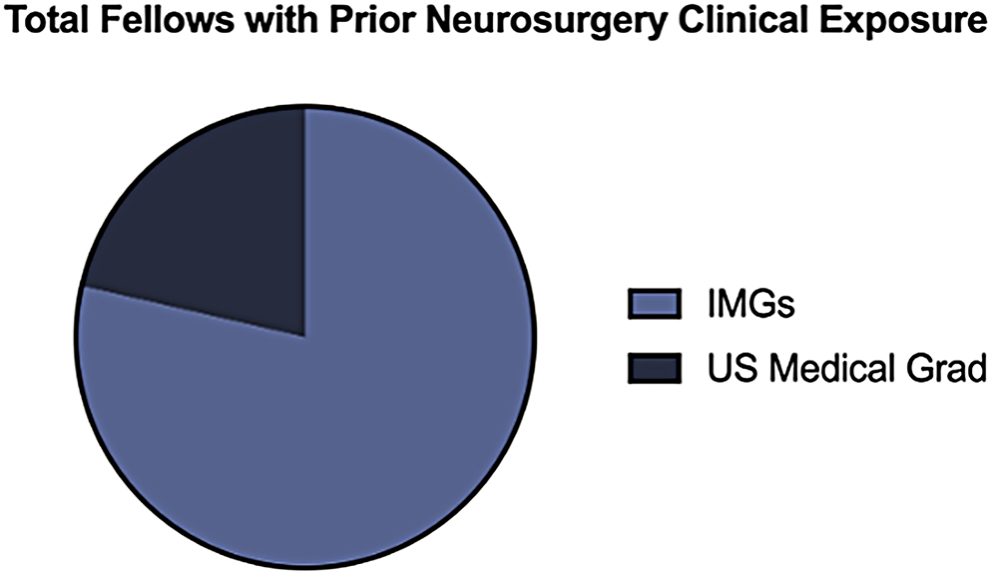
Graph depicting prior neurosurgical clinical exposure prior to acceptance into the pre-residency fellowship program, differentiated between international medical graduates (IMGs) and U.S. medical graduates (*n* = 14)

**Fig. 3:**
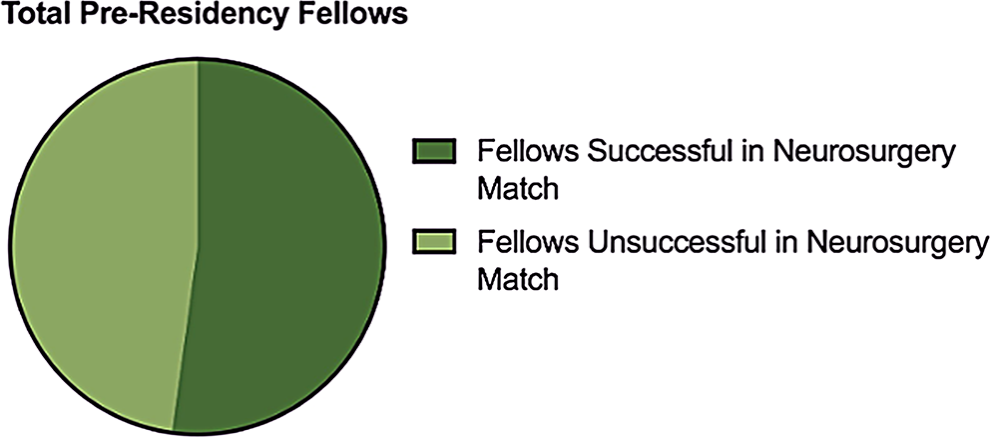
Graph differentiating successful vs. unsuccessful matriculation into neurosurgery residency following graduation from University of Miami’s pre-residency fellowship program (*n* = 23)

In total, 24 individuals who completed a neurosurgical pre-residency fellowship at the University of Miami (UM) from 2000 to 2024 were examined. Of these 24, one individual was excluded due to limited data about their career outcome. A total of 23 fellows were included in the study and ultimately analyzed (Table 1). There was an average of 0.95 fellows per year over the study period of 24 years. Fellows were 65% (*n* = 15) international medical graduates (IMGs) and 35% (*n* = 8) United States (US) medical graduates. 78% (*n* = 18) of total fellows entered the pre-residency fellowship program with prior research experience (Figs. 1) and 61% (*n* = 14) with prior neurosurgical clinical experience outside of medical school exposure (Fig. 2). Out of the 23 fellows that completed their training at UM, 53% (*n* = 12) successfully matched into a US neurosurgery residency (Fig. 3).

**Table 1:** Data of pre-residency fellows at the university of Miami (UM) between 2000 and 2024

	Count (*n*, %)
**Total cohort**	23
**Medical Education**	
United States Medical Graduates	8 (35%)
International Medical Graduates	15 (65%)
**Prior Experiences**	
Research	18 (78%)
Post-Graduate Neurosurgical Exposure	14 (61%)
**Neurosurgery Match Success**	
Successful Match into Neurosurgery	12 (52%)

IMG fellows came from institutions from all over the world, including Turkey, Venezuela, Peru, Spain, Italy, the Dominican Republic, and Saudi Arabia. 6 of 8 US medical graduates that participated in the fellowship did not have a home program.

### Matched fellows

The majority of fellows who matched into neurosurgery residency have been IMGs (75%, *n* = 9). In comparison, 25% (*n* = 3) of total matched fellows have been US medical graduates (Table 2). Only 17% (*n* = 2) of fellows required additional clinical and/or research training after completing the pre-residency fellowship program to match into neurosurgery. Upon analyzing the 12 matched fellows over the study period, 50% (*n* = 6) eventually matriculated to UM for neurosurgery residency. IMGs made up the majority of the UM-matched cohort, at 67% (*n* = 4) compared to 33% (*n* = 2) of US medical graduates. Prior to entering the fellowship program, the majority of matched fellows (58%, *n* = 7) had prior research experience.

**Table 2:** Pre-Residency fellows that successfully matched into neurosurgery

	Count (*n*, %)
**Total Matched Fellows**	12
**Medical Education**	
United States Medical Graduates	3 (25%)
International Medical Graduates	9 (75%)
**Prior Experiences**	
Research	7 (58%)
Post-Graduate Neurosurgical Exposure	8 (67%)
**Post-Fellowship**	
Additional Training Required	2 (17%)
**Total Fellows Matched at UM for Neurosurgery**	6
United States Medical Graduates	2 (33%)
International Medical Graduates	4 (67%)

### International medical graduates

As stated above, most UM pre-residency fellows have been IMGs (65%, *n* = 15) and the majority of these IMGs have successfully matched into neurosurgery (60%, *n* = 9) following completion of the program (Table 3). Out of the 9 who matched, 4 IMGs (44%) went on to complete their residency training at UM. Most IMGs (73%, *n* = 11) had prior neurosurgical clinical exposure through experiences such as prior residency training in their country of origin, attending-level practice, and/or previous US-based pre-residency fellowship training. This contrasts with the 38% (*n* = 3) US medical graduates who entered the fellowship program with neurosurgical clinical experience outside of medical school (Table 4). Additionally, 73% (*n* = 11) IMGs and 88% (*n* = 7) US medical graduates had prior research experience.

**Table 3:** International medical graduate (IMG) pre-residency fellows

	Count (*n*, %)
**Total IMGs**	15
**Prior Experiences**	
Research	11 (73%)
Post-Graduate Neurosurgical Exposure	11 (73%)
**Neurosurgery Match Success**	
Successful Match into Neurosurgery	9 (60%)
Successful Match into UM for Neurosurgery Residency	4 (27%)

**Table 4:** United States medical graduate pre-residency fellows

	Count (*n*, %)
**Total U.S. Medical Graduates**	8
**Medical Education**	
U.S. MD	6 (75%)
U.S. DO	2 (25%)
**Prior Experiences**	
Research	7 (88%)
Post-Graduate Neurosurgical Exposure	3 (38%)
**Neurosurgery Match Success**	
U.S. MD	3 (38%)
U.S. DO	0 (0%)

## Discussion

Matching into neurosurgery residency is a challenging process, with many successful applicants having robust and impressive portfolios [4]. It is also competitive, as 423 US and IMG medical students applied for 241 PGY-1 neurosurgery residency positions in 2024 [1]. In this study, we demonstrate that a pre-residency neurosurgical fellowship has the potential to increase a re-applicant’s competitiveness in obtaining a neurosurgical residency position, specifically if they are an IMG. IMG pre-residency fellows who completed their year-long training with the University of Miami’s neurosurgery department had higher match rates into neurosurgery residency compared to that of US MD graduates (75% vs. 25%). Further, the match rate into neurosurgery residency over the time period analyzed was significantly greater than the national average for IMGs (60% vs. 8%) [2]. However, only 37.5% of US MD graduates who participated in the fellowship matched into a neurosurgical residency. This data suggests that pre-residency neurosurgical fellowship programs are a beneficial pursuit for an IMG but not necessarily for US MDs. It is important to note that a majority of IMG pre-residency fellows who matched from the University of Miami’s program had prior neurosurgical clinical experience. This clearly served as an advantage and could have influenced their success compared to that of US MDs.

Fellowship participants who ultimately matched into neurosurgery, matched primarily at the University of Miami after completing the pre-residency fellowship (50%). These participants were able to demonstrate their clinical knowledge and skills, while forming meaningful relationships within the department that made them assets to the program. Also, throughout the cohort period, the network of IMGs graduating from the pre-residency program expanded which allowed for more IMGs to learn about the program through their peers and apply, contributing to the higher volume of IMG participants. 5 out of the 9 successful IMG pre-residency fellows matched elsewhere other than the University of Miami. The geographic regions of their match locations were all over the country, excluding the west coast of the US, from the University of Oklahoma to New York Medical College. One IMG who matched at the University of Buffalo was a prior general surgery resident at Mount Sinai, which could have influenced their decision to train in this region. Of the 6 IMGs who did not match at University of Miami, common themes were slightly lower STEP 2 scores, matching at different institutions, and/or matching into a different specialty. Performance in the program was also a factor in deciding to match a fellowship participant or not. However, due to fluctuation of department members who conducted residency interviews for these IMG applicants throughout the cohort period, we cannot define any particular unsuccessful criteria that inhibited an IMG from matching at University of Miami. Matching into neurosurgery overall is dependent on multiple factors for any applicant, IMG or US MD, and the pre-residency fellowship provides key experiences to increase competitiveness. Data regarding the outcomes of fellows with unsuccessful re-application is limited, however some pursuits include medical consulting, transition into general surgery, and return to their country of origin to practice neurosurgery.

To be successful, an applicant usually has ample research experience [4]. A survey in 2018 performed by the NRMP showed that 76% of program directors cited research as an important factor in selecting candidates to interview [4]. Many program director’s view a medical student’s research productivity in the neurosurgical field as proof of dedication to the specialty and their ability to see a project from its inception to its completion [5]. Furthermore, a study by Hulou et al. demonstrated that the number of pre-residency publications correlated significantly with a successful match to a top 25 residency program [6]. They also saw a correlation in the number of publications pre-residency to the number of publications intra-residency [6].

Some applicants into the field of neurosurgery may be at a disadvantage due to a small neurosurgery department or lack of a home program in its entirety [7]. Interestingly, IMGs seem to have an advantage over MD seniors applying to neurosurgery in this regard. One study surveyed neurosurgery program directors throughout the country and reported that they expected applicants to spend 12–24 months on research before applying [8]. IMGs that were surveyed reported spending on average 30 months doing research with a median of 12 publications before submitting their application [8]. 22 program directors also reported that they believed IMGs completed more research before submitting their residency application compared to US MD students. The data described here implies that pre-residency fellowship programs allow US medical graduates who are planning to re-apply into neurosurgical residency to broaden their research portfolio and make meaningful contributions to the field, all while increasing the competitiveness of their application. Additionally, although IMGs typically have completed more research than US medical graduates, these programs allow these students to demonstrate their research capabilities in a US academic setting.

Previously, STEP 1 had been a large factor in evaluating candidates for neurosurgical residency [9]. However, a survey conducted by Stein et al. surveying 117 neurosurgery residency programs, found that although STEP 1 is now binary, program directors will still rely on objective measurements when evaluating candidates [10]. The authors of this study believe a higher amount of emphasis will be placed on STEP 2 CK scores, with the results of their study reflecting the largest increase in median importance of all the factors they studied [10]. Interestingly, program directors who were respondents of the study implicated that both STEP 1 and STEP 2 CK examinations were worse predictors of residents’ clinical performance and their ability to pass the American Board of Neurological Surgeons examination [10]. Medical schools also give different amounts of time to prepare for STEP 2 CK, which can lead to unexpected inequalities when evaluating potential neurosurgery residency candidates based on this merit [10]. IMGs must prepare and take both of these examinations before being able to practice any kind of medicine in the United States. Still, even if an IMG student scored > 260 on STEP 2 CK their chance of successfully matching into neurosurgery are ~ 55% [2]. This analysis implies that participation in a pre-residency fellowship program gives an MD re-applicant a chance to stand out from their peers clinically and prove that they are capable of more than what their STEP 2 CK scores may identify. Furthermore, IMGs who score well but are overlooked have the opportunity to build a relationship with programs and prove their clinical expertise in real-time.

Another large factor that neurosurgery programs observe when considering a residency applicant is the prestige of the medical school at which they attend. Traditionally, NIH funding has been used as a proxy for medical school prestige in the context of neurosurgery match [11]. A study by Kortz et al. analyzed how medical school prestige played into the consideration of MD seniors’ applications into neurosurgery programs. They found that schools with more NIH funding placed more students into neurosurgery residency compared to schools with less funding [11]. The authors of this study also note this could be related to research productivity, as students at schools with greater NIH funding had more research productivity defined by peer-reviewed work [11]. MD seniors pursuing neurosurgery residency from less prestigious medical schools are put at a disadvantage in this regard, but even more-so, IMG applicants who often have no access to institutions with NIH funding are critically affected by the implication of this data. The data demonstrated here indicate pre-residency neurosurgery fellowship programs, especially at institutions with ample NIH funding, can mitigate this potential effect on their application and improve their research output.

Lastly, a the size and quality of an applicant’s network in the field is important when it comes to match success. Typically, 4th year medical students will complete acting internships at different institutions throughout the country to increase the number of people that they know in the field and show their capabilities [12]. This allows students to receive letters of recommendation, which are highly valued in the neurosurgery match. A study by Mulligan et al. analyzing the 2021 match described this process as critical to a successful match into neurosurgery [12]. Students also typically complete an acting internship at their home program if available [12]. Even for students who do not have a home program, the Society for Neurological Surgeons developed a cohesive national curriculum that allows for students’ to find an “adoptive” home program that they hope decreases inequity within the field of neurosurgery [12]. Specifically at a disadvantage are IMGs in this regard, as they infrequently have the opportunity to make meaningful connections and gain letters of recommendation before applying. However, there are opportunities being created to assist US medical students in developing their neurosurgery network. For example, The University of Miami’s neurosurgery program created the initiative “Medical Student Summer Research Scholarship in Neurosurgery” to help students develop their network within the field. This past year, the program had 14 different academic neurosurgery programs, and 25 scholars participated [7]. This program develops a students’ curriculum vitae through research participation, meeting pertinent faculty and residents, and acquiring unique clinical experiences to increase their competitiveness to match into neurosurgery when the time comes [7].

This study has several limitations. The small sample size of 23 fellows requires some caution when interpreting results. However, to date, there are less than 10 neurosurgery pre-residency fellowship programs in the country. Each of these programs accepts only a few candidates per year.The cohort described here, although small, is likely the largest out of existing neurosurgery pre-residency fellowship programs. That being said, we acknowledge the disproportionate amount of those fellows described here matching into neurosurgical residency at University of Miami and recognize our data does not necessarily represent other departmental experiences.There are also no current studies to-date that directly evaluate and compare neurosurgery match success among reapplicant pre-residency fellows and reapplicant ACGME general surgery preliminary residents. Such data would be extremely beneficial when critically assessing the benefits of this program compared to other avenues. With that said, University of Miami pre-residency fellows have reported better success in matching into neurosurgery (52%) compared to reapplicant ACGME general surgery preliminary residents (25–33%) [13] We expect that as more programs emerge, further trends in competitive factors and participant outcomes can be explored. There is also a risk of bias as the authors are primarily affiliated with UM’s Department of Neurosurgery. Lastly, the authors frequently compare differences in success among IMGs and US medical graduates, however the sample sizes are not equal in these two groups. Future directions include continued analysis of the program in subsequent years, external review, and comparison of the University of Miami’s program to other pre-residency fellowship programs.

## Conclusion

In this study, we analyzed the pre-residency neurosurgery fellowship program at the University of Miami and found that the majority of participants in the program matched successfully into neurosurgery. Of these fellows, just under half were eventually accepted into neurosurgery residency at the University of Miami. The results of this study are important to disseminate so that unmatched applicants have an opportunity to learn about the outcomes of participating in a program such as this. Furthermore, IMGs can be provided with the opportunity to interact with US program directors, faculty, and residents, as well as learn about the outcomes of their IMG colleagues that completed the University of Miami’s program. By sharing this data, we hope that other institutions with pre-residency neurosurgery fellowship programs will conduct their own analyses, so that we may continue to recruit providers with a diverse array of expertise, experiences, and talent.

## Data Availability

No datasets were generated or analysed during the current study.

## References

[1] https://www.nrmp.org/wp-content/uploads/2024/03/Advance-Data-Tables-2024.pdf. https://www.nrmp.org/wp-content/uploads/2024/03/Advance-Data-Tables-2024.pdf. Accessed 30 Jun 2024

[2] Yekula A, Sreeram S, Dhawan S et al (2024) Neurosurgery residency match for international medical graduates in the united States. J Neurosurg 140:291–298. 10.3171/2023.5.JNS23556 37548564 10.3171/2023.5.JNS23556

[3] Lu VM, Chambless LB (2020) International medical graduates applying to neurosurgical residency in the united States through the lenses of an applicant versus a program director. World Neurosurg 142:299–300. 10.1016/j.wneu.2020.07.046 32683004 10.1016/j.wneu.2020.07.046

[4] Price G, Lakomkin N, Kamat S et al (2021) Medical student publications in neurosurgery: at which U.S. Academic institutions do medical students publish most? World Neurosurg 147:181–189e1. 10.1016/j.wneu.2020.12.045 33338672 10.1016/j.wneu.2020.12.045

[5] Karsy M, Henderson F, Tenny S et al (2018) Attitudes and opinions of US neurosurgical residents toward research and scholarship: a National survey. J Neurosurg 131:252–263. 10.3171/2018.3.JNS172846 30117774 10.3171/2018.3.JNS172846

[6] Hulou MM, Samaan CA, McLouth CJ et al (2023) Competitive neurosurgery residency programs: predictors of matching outcome and research productivity. Clin Neurol Neurosurg 232:107884. 10.1016/j.clineuro.2023.107884 37467577 10.1016/j.clineuro.2023.107884

[7] Klein BR, Sowah MN, Levi AD (2023) The role of limited access to students from more diverse nonfeeder medical schools in creating diversity inequities in neurosurgical residency. Neurosurg Focus 55:E13. 10.3171/2023.8.FOCUS23458 37913545 10.3171/2023.8.FOCUS23458

[8] Mignucci-Jiménez G, Xu Y, Houlihan LM et al (2022) Analyzing international medical graduate research productivity for application to US neurosurgery residency and beyond: A survey of applicants, program directors, and institutional experience. Front Surg 9:899649. 10.3389/fsurg.2022.899649 35965866 10.3389/fsurg.2022.899649 PMC9363657

[9] Yu N, Hoch JS, Martin AR, Shahlaie K (2023) Trends in successfully matched neurosurgery residency applicants. J Neurosurg 1–7. 10.3171/2023.3.JNS222397 10.3171/2023.3.JNS222397 37086164

[10] Stein JS, Estevez-Ordonez D, Laskay NMB et al (2022) Assessing the impact of changes to USMLE step 1 grading on evaluation of neurosurgery residency applicants in the united States: A program director survey. World Neurosurg 166:e511–e520. 10.1016/j.wneu.2022.07.045 35843584 10.1016/j.wneu.2022.07.045

[11] Kortz MW, McCray E, Strasser T et al (2021) The role of medical school prestige and location in neurosurgery residency placement: an analysis of data from 2016 to 2020. Clin Neurol Neurosurg 210:106980. 10.1016/j.clineuro.2021.106980 34673366 10.1016/j.clineuro.2021.106980

[12] Mulligan KM, Pan X, Gerges C et al (2022) The 2021 neurosurgery match: an analysis of the impact of virtual interviewing and other COVID-19-Related changes. World Neurosurg 162:e8–e13. 10.1016/j.wneu.2021.11.109 34864190 10.1016/j.wneu.2021.11.109 PMC8989630

[13] Agarwal N, Reddy V (2021) Surviving neurosurgery: vignettes of resilience. Springer International Publishing, Cham

